# Evidence construction of Silibinin capsules against alcoholic liver disease based on a meta-analysis and systematic review

**DOI:** 10.3389/fphar.2025.1516204

**Published:** 2025-02-04

**Authors:** Yan Wang, Wenmin Yang, Yuan Yang, Xingning Liu, Lanfen Peng, Qi Huang, Kongli Fan, Rui Hu, Jinyu Yi, Xin Zhong, Jing Li, Jialing Sun, Xiaozhou Zhou

**Affiliations:** ^1^ Department of Liver Disease, The Fourth Clinical Medical College of Guangzhou University of Chinese Medicine, Shenzhen, China; ^2^ Department of Liver Disease, Shenzhen Traditional Chinese Medicine Hospital, Shenzhen, China; ^3^ Faculty of Chinese Medicine, Macau University of Science and Technology, Taipa, Macao, China

**Keywords:** Silibinin capsules, ALD, rct, meta-analysis, systematic review, Chinese patent

## Abstract

**Background:**

In recent years, the incidence of alcoholic liver disease (ALD) has rapidly increased worldwide, becoming a significant health issue. Silibinin capsules have shown potential in treating ALD, but clinical evidence is still insufficient. This meta-analysis aimed to evaluate the efficacy and safety of Silibinin capsules in the treatment of ALD.

**Methods:**

The study was registered with PROSPERO (CRD42024509676). Randomized controlled trials (RCTs) were included from six databases, covering the period from database inception to 30 December 2023. Primary outcomes included liver function indicators such as alanine aminotransferase (ALT), aspartate aminotransferase (AST), gamma-glutamyl transferase (GGT), total bilirubin (TBIL), lipid indicators including triglycerides (TG) and total cholesterol (TC), coagulation indicators including prothrombin time (PT), liver fibrosis indicator (PC-III), and Effective Rate. Analysis was performed using Review Manager 5.4.1 and STATA 14.0.

**Results:**

In 15 RCTs involving 1,221 patients, compared to the non-Silibinin group, Silibinin capsules showed significant efficacy in terms of liver function, lipid levels, and effective rate in patients with ALD. Detailed parameters were as follows: ALT [SMD = −1.16, 95% CI (−1.84, −0.47)], AST [SMD = −1.56, 95% CI (−2.18, −0.95)], GGT [SMD = −1.48, 95% CI (−2.09, −0.87)], TBIL [SMD = −1.14, 95% CI (−2.16, −0.13)], TG [SMD = −1.29, 95% CI (−1.93, −0.66)], TC [SMD = −1.11, 95% CI (−1.61, −0.61)], PT [SMD = −0.01, 95% CI (−0.29, 0.26)], PC-III [SMD = −1.94, 95% CI (−3.04, −0.84)], and Effective Rate [OR = 3.60, 95% CI (2.28, 5.70)]. Importantly, Silibinin capsules exhibited a favorable safety profile, with only mild gastrointestinal reactions and reports of insomnia as adverse events.

**Conclusion:**

This review reveals the clinical efficacy and safety of Silibinin capsules in the treatment of ALD, and confirms that the drug is an effective adjuvant therapy to alleviate ALD. At present, the mechanism of action of this drug for ALD is still unclear, and we expect more experimental studies to prove the clinical value of Silibinin capsules.

**Systematic Review Registration:**

https://www.crd.york.ac.uk/PROSPERO/display_record.php?RecordID=509676.

## 1 Background

Alcoholic liver disease (ALD) is a serious condition that develops as a result of prolonged and excessive alcohol consumption. There are different stages of ALD, including alcoholic fatty liver, alcoholic hepatitis with fibrosis, alcoholic cirrhosis, and in severe cases, which can lead to the development of hepatocellular carcinoma (HCC) (Asrani et al., 2019). ALD can significantly impact liver function and overall health, making it important for individuals to recognize the risks associated with excessive alcohol intake. Furthermore, it is essential for individuals with ALD to seek medical attention and treatment to prevent further progression of the disease and potential complications such as liver failure or liver cancer. Lifestyle changes, such as reducing alcohol consumption and maintaining a healthy diet, can also help manage ALD and improve overall liver health. By raising awareness about the dangers of excessive alcohol consumption and promoting early intervention and treatment, we can work towards reducing the burden of ALD on individuals and healthcare systems.

Since 2014, the prevalence of ALD has been increasing, especially in recent years, with a rapid acceleration following the COVID-19 pandemic where ALD mortality increased by 24.8%, resulting in significant healthcare and economic burdens. Early-stage ALD may be asymptomatic, characterized by fat degeneration or alcoholic steatohepatitis and early fibrosis (Kuo et al., 2023). Late-stage ALD encompasses a range of serious complications such as cirrhosis, ascites, hepatic encephalopathy, variceal hemorrhage, hepatocellular carcinoma, and acute or chronic liver failure. The risk of death within 1 month for individuals with late-stage ALD is reported to be between 20% and 50% (Jophlin et al., 2024).

Current treatment approaches for ALD include abstinence from alcohol, nutritional support, medication, and surgical interventions. Alcohol consumption exacerbates liver disease progression in patients with obesity and metabolic syndrome, with abstinence being a key determinant of long-term outcomes (Chang et al., 2020). Medications such as corticosteroids and acamprosate are used but often have multiple side effects and limited efficacy. Liver transplantation is the ultimate treatment option for advanced cirrhotic patients. Recently, numerous studies have indicated that various natural compounds exhibit a beneficial therapeutic impact on diseases. In particular, herbal extracts have been highlighted for their considerable importance in treating ALD. These findings underscore the potential of plant-derived substances in medical treatments, offering a promising alternative or complement to conventional therapies (Singal and Mathurin, 2021).

Silymarin, a flavonolignan compound derived from the milk thistle herb (Silybum marianum), has demonstrated a critical role in the management and treatment of liver diseases, blood-related disorders, arthritis, ulcerative colitis, and cancer pathologies (Akhtar et al., 2023). A recent study found that silymarin is also effective in relieving symptoms of depression and anxiety. Silymarin is the main active component of Silybum marianum and it includes silybin (silibinin), isosilybin, silydianin, and silychristin (Ferreira et al., 2017). Silibinin, which has the molecular formula of C_25_H_22_O_10_, is an important active ingredient of the flavonoid lignan mixture silymarin. It has been confirmed to exert the pharmacological effect of protecting hepatocytes by improving the stability of hepatocyte membranes, antioxidant, antifibrotic, anti-inflammatory and other pharmacological pathways. Due to its chemical structure, it is a difficult soluble drug, soluble in acetone, ethyl acetate, ethanol, slightly soluble in chloroform, insoluble in water, with low solubility and poor lipid solubility, and its poor bioavailability limits its clinical use. Therefore, in order to improve the bioavailability of silibinin, scholars developed Silibinin capsules (trade name “Shui Linjia”), which are silibinin phospholipid complexes, each capsule contains 35 mg of silibinin and 65 mg of phospholipids, and the indications are used for the recovery of abnormal liver function in acute and chronic hepatitis and fatty liver (Křen and Valentová, 2022). Silibinin capsules reduces fat accumulation and oxidative imbalance in steatotic cells by decreasing the levels of reactive oxygen species (ROS), lipid peroxidation, and catalase activity, while also mitigating the inflammatory response and NF-κB activation. It inhibits NF-κB in Kupffer cells and facilitates hepatocyte regeneration pathways, thereby suppressing the progression of alcoholic fatty liver. Silibinin also inhibits TGF-β1 mRNA and activates hepatic stellate cells (Detaille et al., 2008; Yoo et al., 2004). Although the ingredients of Silibinin capsules are derived from plants, they are processed through chemical processing and formulation techniques into capsules, conforming to the characteristics of western medicine. However, current clinical studies on Silibinin capsules for ALD treatment vary in design and quality, lacking robust evidence of its clinical efficacy and safety. The potential mechanisms of Silibinin in treating ALD also require further clarification.

In summary, these findings suggest that Silibinin may inhibit mechanisms related to lipid metabolism, fibrosis, and sclerosis in ALD patients, potentially slowing disease progression. Meta-analysis, by aggregating a large body of research, increases sample size, enhances accuracy, and strengthens statistical power, thus providing more convincing conclusions. It can also quantify and carefully examine differences in results across studies, helping to identify whether inconsistencies are due to methodological heterogeneity or data limitations, such as small sample size, gender differences, narrow age range, or limited experimental conditions. Therefore, this study seeks to collect medical literature on the treatment of silibinin in ALD, utilizing the Cochrane system to comprehensively and objectively evaluate its clinical efficacy and safety, providing evidence-based support for its clinical use.

## 2 Methods

This systematic review complies with the PRISMA (Preferred Reporting Items for Systematic Reviews and Meta-Analyses) 2020 Statement (Page et al., 2021), which was registered with PROSPERO (CRD42024509676).

### 2.1 Search strategy

In this study, several databases were utilized to conduct a comprehensive search for relevant literature. These databases included Wanfang Data, Chinese National Knowledge Infrastructure, VIP Medicine Information System, PubMed, Embase, and Web of Science, among others, to search for randomized controlled trials (RCTs) on Silibinin treatment for ALD. The search period extended from the inception of the databases to 30 December 2023, with language restrictions to Chinese and English. The search terms included (“Silibinin” or “Silybin” or “Silybinin” or “Silibin” or “Ardeyhepan” or “Cefasilymarin” or “Durasilymarin” or “Hepa-loges” or “Hepa loges” or “Hepa-Merz Sil” or “Hepa Merz Sil” or “Silybin B” or “Silibinin B” or “Alepa-forte” or “Alepa forte” or “Hepar-Pasc” or “Hepar Pasc” or “Heparsyx” or “Lagosa” or “2,3-Dehydrosilybin” or “2,3 Dehydrosilybin” or “Legalon Forte” or “Silybin A” or “Silibinin A” or “Heplant”) AND (“Alcoholic Liver Diseases” or “Alcoholic Liver Disease” or “Liver Disease, Alcoholic” or “Liver Diseases, Alcoholic”). The search strategy was structured accordingly.

Two researchers independently conducted searches using specific keywords in different databases. Subsequently, the results were compared and analyzed to ensure completeness and accuracy, with any discrepancies resolved through consensus.

### 2.2 Inclusion and exclusion criteria

The criteria for inclusion were: (1) Type of study: based on randomized controlled trial (RCT) methodology; (2) Type of subjects: individuals diagnosed with ALD; (3) Type of intervention: experimental group receiving Silibinin or combined treatment with Silibinin and other drugs; (4) Type of outcomes: transaminase (ALT, AST, GGT), TBIL, lipid indicators (TG, TC), coagulation indicators (PT), liver fibrosis indicators (PC-III) and Effective Rate.

The following types of articles were excluded: animal experiments, reviews, case reports, conference abstracts, duplicate publications, incomplete or non-compliant articles, and non-RCT literature.

### 2.3 Data collection and extraction

Data collection and extraction were carried out separately by two evaluators, with discrepancies resolved by a third researcher. The information gathered from the selected studies encompassed several critical details, including first author, year of publication, sample size, respective number of experimental and control groups, intervention method, and trial duration. Outcome measures included ALT, AST, GGT, TBIL, TG, TC, PT, PC-III, and Effective Rate. For continuous variables reported as medians with interquartile ranges, mean ± standard deviation was calculated using validated mathematical methods. Missing or unreported data were obtained by contacting the respective authors.

### 2.4 Methodological quality assessment

Two researchers independently assessed bias risk using the Cochrane Collaboration’s tool (Higgins et al., 2011). Methodological quality was evaluated across seven dimensions to clarify bias and reliability: random sequence generation, allocation concealment, blinding of participants and personnel, blinding of outcome assessment, incomplete outcome data, selective reporting, and other biases. Quality and level of evidence for eligible studies were independently assessed by two reviewers, with any differences resolved through discussion.

### 2.5 Data synthesis and statistical analysis

Meta-analyses were performed using RevMan 5.4.1 software, with relative risk (RR) and 95% confidence interval (CI) for dichotomous variables, and standardized mean difference (SMD) and 95% CI for continuous variable data. Heterogeneity was assessed using the chi-square test, with a fixed-effects model (P ≥ 0.05 or I^2^ ≤ 50%) when there was no heterogeneity or low heterogeneity, and a random-effects model (P < 0.05 or I^2^ > 50%) for high heterogeneity. When results showed significant heterogeneity, subgroup analyses were conducted to identify sources of heterogeneity based on the level of Silibinin intake, intervention duration, and other factors. Sensitivity analyses were performed to reveal the stability of the findings by excluding each study of lower quality.

### 2.6 Assessment of publication bias

Publication bias was examined through the use of funnel plots generated in Review Manager 5.4.1. Additionally, Egger’s regression test (Egger et al., 1997) in Stata 14.0 was applied to conduct a statistical assessment of publication bias, considering a significance level of P < 0.05. These tests were crucial in determining the presence of any potential publication biases in the data analysis.

## 3 Results

### 3.1 Literature selection

**FIGURE 1:**
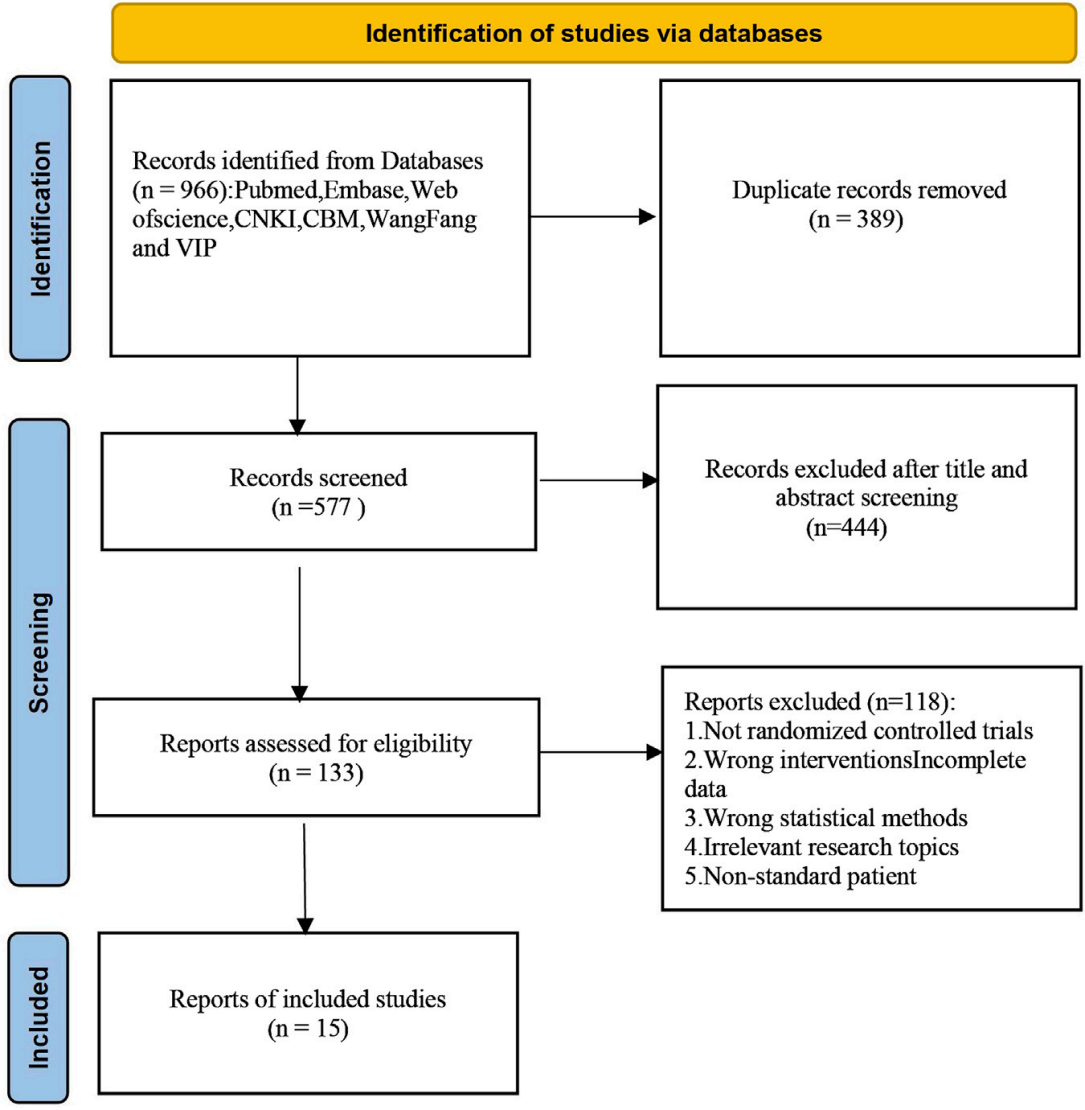
Flow chart.

A total of 966 articles were initially retrieved, of which 389 duplicates were removed after checking. After reviewing titles and abstracts, 562 irrelevant articles were excluded. These exclusions included articles that did not meet the criteria for RCTs, did not involve ALD patients, or lacked comprehensive data on Silibinin use. Following the inclusive and exclusive criteria, a total of 15 articles were included. The included RCTs were conducted in China, Hungary, and Austria, with the earliest study published in 1988. The literature selection process is illustrated in Figure 1.

### 3.2 Study characteristics

This study included 15 RCTs conducted between the inception of the database and 2023, involving 1,221 patients. Among these studies, 13 RCTs were conducted in China, while 2 were conducted in Hungary and Austria. In 13 RCTs, the control group received hepatoprotective drugs, while in the remaining 2 RCTs, the control group was given a placebo. Among the 15 RCTs, six studies had a daily intake of Silibinin between 200–400 mg, and six studies had a daily intake greater than 400 mg. One study had a daily intake of Silibinin less than 200 mg, and two studies did not mention the dose of Silibinin. The duration of Silibinin intervention ranged from 3 weeks to 48 weeks. Seven RCTs provided diagnostic criteria for ALD, while the remaining eight RCTs did not provide diagnostic criteria for ALD. The characteristics of each included study are shown in Table 1.

**TABLE 1:** Study characteristics of included studies.

Study	Sample size	Sex	Age		Duration (months)	Measurements		Dose of drug	Outcomes
	(Treatment group/Control group)	(Male/Female)	Treatment group	Control group		Treatment group	Control group		
Chen (2014)	42/40	82/0	36.8 ± 5.6	37.2 ± 5.7	2	Glutathione + Silymarin	Glutathione	0.4 g, tid	1,2,9
Fu (2013)	43/36	67/42	20–60	20–60	3	Conventional therapy + Silibinin capsules	Conventional therapy + Hepatology Capsules	0.75, tid	1,2,3,5,6,9
Guo et al. (2010)	38/22	54/6	26–58	26–58	2	Silibinin capsules	Xuezhikang Capsules	0.70, tid	1,2,3,5,6,9
Han et al. (2011)	56/52	108/0	26–58	26–58	1	Silibinin capsules + Polyene Phosphatidylcholine + Nutritional therapy	Polyene Phosphatidylcholine + Nutritional therapy	0.4 g, tid	2,3,4,7.9
Jie et al. (2012)	34/34	68/0	22–65	22–65	1	Silymarin + Glutathione + Nutritional therapy	Glutathione + Nutritional therapy	0.14 g, bid	1,2,3,4,8,9
Li and Liu (2016)	30/23	47/19	44.1 ± 2.6	43.9 ± 2.2	0.75	Conventional therapy + Silibinin capsules	Conventional therapy + Hepatology Capsules	0.14 g, tid	9
Liu et al. (2012)	45/45	77/13	38.6 ± 12.4	38.6 ± 12.4	3	Silibinin capsules	Glutathione	0.14,tid	1,2,3,5,6,9
Liu et al. (2018)	36/32	58/18	58.9 ± 6.8	58.7 ± 6.9	12	Conventional therapy + Silibinin capsules	Conventional therapy	0.70 g, bid	1,2,8,9
Quan et al. (2018)	40/40	57/23	47.5 ± 4.3	47.5 ± 4.3	1.5	Conventional therapy + Silibinin capsules	Conventional therapy + Hepatology Capsules	0.14 g, tid	1,2,3,5,6,9
Tian and Han (2015)	30/30	60/0	25–70	25–70	3	Glutathione + Silymarin	Glutathione	0.14 g, tid	1,2,5,6,9
Yin and Yang (2009)	60/20	NA	22–60	24–59	3	Silibinin capsules	ZhiBiTuoPian	0.70 g, tid	1,2,9
Zhou et al. (2008)	30/29	56/3	44 ± 8	42 ± 7	6	Silymarin capsules	Hu Gan Pian	0.14 g, tid	1,2,3,4,5,7,8
Zhu et al. (2003)	40/20	60/0	27–56	25–60	3	Silibinin capsules	Hu Gan Pian + ZhiBiTuoPian	0.70 g, tid	1,2,9
Fehér et al. (1998)	17/19	27/9	38 ± 7	44 ± 6	6	Silymarin	placebo	0.14 g, tid	NA
Ferenci et al. (1989)	45/47	NA	57 ± 12	58 ± 12	2	Silymarin	placebo	0.14 g, tid	1,7

1, ALT; 2, AST; 3, GGT; 4, TBIL; 5, TG; 6, TC; 7, PT; 8, PC-III; 9, Effective Rate.

### 3.3 Evaluation of potential bias

**FIGURE 2:**
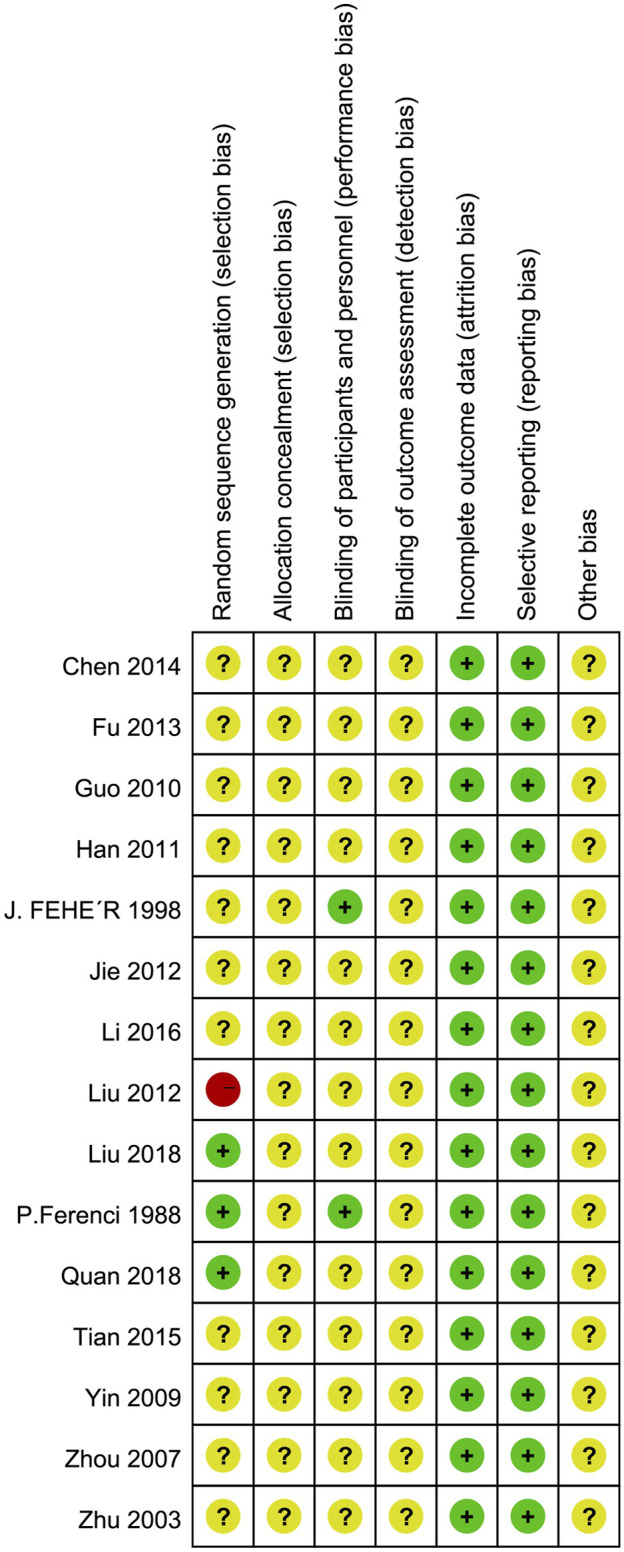
Assessment risks of bias. “+” = low risk of bias, “?” = unclear risk of bias, and “−” = high risk of bias.

The findings of the evaluation of potential bias for the 15 studies were illustrated in Figure 2. Three RCTs explicitly described randomization using a random number table, classified as “low risk” assessment. One RCT used visit sequence for grouping, considered pseudo-random, and assessed as “high risk” bias. One RCT described random generation but did not specify the correctness of the random method, rated as “unclear” bias. Allocation concealment was unclear for all included RCTs. Two RCTs mentioned blinding of participants, while blinding of outcome assessors and researchers was unclear for all other RCTs, rated as “unclear” bias. The included RCTs assessed objective indicators such as ALT, AST, GGT, TC, TG, etc., categorized as “low risk” for detection bias. Other biases were rated as unclear.

### 3.4 Effects of Silibinin on liver functions

**FIGURE 3:**
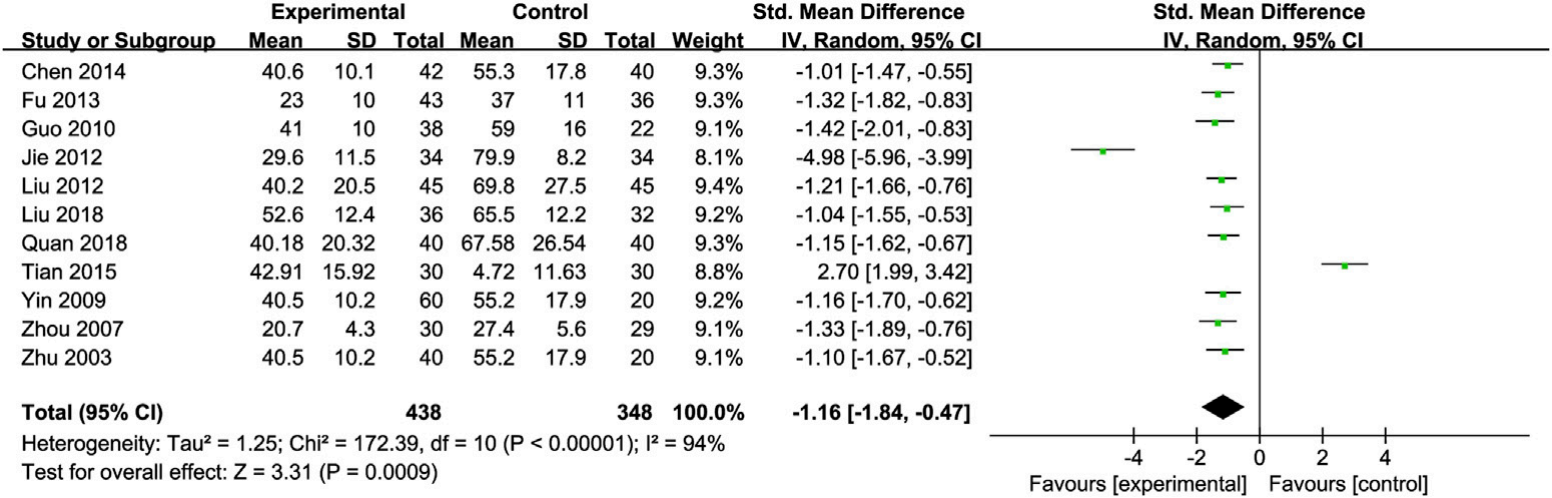
Forest plot of ALT.

Alanine Transaminase (ALT): Eleven RCTs (Chen, 2014; Fu, 2013; Guo et al., 2010; Jie et al., 2012; Liu et al., 2012; Liu et al., 2018; Quan et al., 2018; Tian and Han, 2015; Yin and Yang, 2009; Zhou et al., 2008; Zhu et al., 2003) involving 438 ALD patients evaluated ALT levels. The meta-analysis demonstrated a notable decrease in alanine aminotransferase (ALT) levels when treated with Silibinin. The standardized mean difference (SMD) was −1.16 with a 95% CI ranging from −1.84 to −0.47, and this result was statistically significant (P = 0.0009). Additionally, the analysis showed a high level of heterogeneity, as indicated by an I^2^ value of 94%, which was illustrated in Figure 3. Subgroup analysis based on Silibinin intervention duration >3 months showed significantly reduced heterogeneity (I^2^ = 0%) (Table 2).

**TABLE 2:** Subgroup analysis for outcomes.

	Number of comparisons	SMD [95% CI]	P-value for overall effect	I^2^ (%)	P-value for subgroup differences
ALT					
Daily intake of Silibinin(g)					
<0.20	1	−1.04 [−1.55, −0.53]	<0.0001	NA	0.16
0.20–0.40	6	−1.74 [−2.52, −0.97]	<0.0001	91	
>0.40	4	−0.26 [−1.82, 1.29]	0.74	97	
Total intake of Silibinin (g)					
<25	7	−1.64 [−2.28, −1.00]	<0.00001	89	0.27
25–50	3	0.14 [−1.99, 2.26]	0.90	98	
>50	1	−1.33 [−1.89, −0.76]	<0.00001	NA	
Duration of Silibinin intervention (months)					
<3	4	−2.06 [−3.29, −0.83]	0.001	94	0.98
= 3	5	−0.44 [−1.67, 0.79]	0.48	96	
>3	2	−1.17 [−1.54, −0.79]	<0.00001	90	
AST					
Daily intake of Silibinin (g)					
<0.20	1	−0.94 [−1.37,-0.50]	<0.0001	NA	0.09
0.20–0.40	6	−1.69 [−2.43,-0.95]	<0.00001	90	
>0.40	4	−2.03 [−3.23,-0.82]	0.0010	94	
Total intake of Silibinin (g)					
<25	7	−1.56 [−2.19,-0.93]	<0.00001	89	0.61
25–50	3	−1.99 [−3.52,-0.46]	0.01	95	
>50	1	NA	NA	NA	
Duration of Silibinin intervention (months)					
<3	5	−1.49 [−2.72,-0.27]	0.02	96	0.28
=3	5	−1.61 [−2.41,-0.80]	<0.0001	91	
>3	2	−1.65 [−2.95, −0.34]	0.01	0	
GGT					
Daily intake of Silibinin (g)					
<0.20	1	−1.13 [−1.58, −0.69]	<0.0001	NA	0.23
0.20–0.40	3	−2.00 [−2.92,-1.07]	<0.0001	86	
>0.40	2	−1.52 [−2.29,-0.74]	0.0001	74	
Total intake of Silibinin (g)					
<25	4	−1.77 [−2.53, −1.02]	<0.00001	86	0.08
25–50	1	−1.13 [−1.58, −0.69]	<0.00001	NA	
>50	1	−1.94 [−2.57, −1.31]	<0.00001	NA	
Duration of Silibinin intervention (months)					
<3	4	−1.27 [−2.15, −-0.38]	0.005	94	0.48
=3	2	−1.70 [−2.83, −0.57]	0.003	25	
>3	1	−1.94 [−2.57, −1.31]	<0.00001	NA	
TBIL					
Daily intake of Silibinin (g)					
<0.20	0	NA	NA	NA	0.67
0.20–0.40	1	−2.05 [−2.65, −1.46]	<0.00001	NA	
>0.40	1	−2.25 [−2.91, −1.59]	<0.00001	NA	
Total intake of Silibinin (g)					
<25	1	−2.05 [−2.65, −1.46]]	<0.00001	NA	0.67
25–50	0	NA	NA	NA	
>50	1	−2.25 [−2.91, −1.59]	<0.00001	NA	
Duration of Silibinin intervention (months)					
<3	2	−2.25 [−2.91, −1.59]	0.39	97	0.76
= 3	0	NA	NA	NA	
>3	2	−1.37 [−3.04, 0.31]	0.11	0	
TG					
Daily intake of Silibinin (g)					
<0.20	0	NA	NA	NA	0.57
0.20–0.40	2	−1.58 [−3.15, −0.02]	0.05	94	
>0.40	3	−1.09 [−1.73, −0.46]	0.0008	0	
Total intake of Silibinin (g)					
<25	3	−1.24 [−2.10, −0.38]	0.005	90	0.56
25–50	2	−0.97 [−1.28, −0.65]	<0.00001	40	
>50	0	NA	NA	NA	
Duration of Silibinin intervention (months)					
<3	2	−0.76 [−1.11, −0.41]	<0.0001	0	0.10
= 3	3	−1.66 [−2.67, −0.65]	0.001	91	
>3	0	NA	NA	NA	
TC					
Daily intake of Silibinin (g)					
<0.20	0	NA	NA	NA	0.87
0.20–0.40	2	−1.06 [−1.85, −0.28]	0.008	78	
>0.40	3	−1.16 [−1.95, −0.36]	0.004	87	
Total intake of Silibinin (g)					
<25	3	−0.98 [−1.45, −0.51]	<0.0001	63	0.62
25–50	2	−1.36 [-2.81, 0.09]	0.07	93	
>50	0	NA	NA	NA	
Duration of Silibinin intervention (months)					
<3	2	−0.75 [−1.10, −0.40]	<0.0001	0	0.17
=3	3	−1.38 [−2.21, −0.55]	0.001	87	
>3	0	NA	NA	NA	
PT					
Daily intake of Silibinin (g)					
<0.20	0	NA	NA	NA	0.67
0.20–0.40	1	−2.05 [−2.65, −1.46]	<0.00001	NA	
>0.40	1	−2.25 [−2.91, −1.59]	<0.00001	NA	
Total intake of Silibinin (g)					
<25	0	NA	NA	NA	NA
25–50	0	NA	NA	NA	
>50	1	−0.25 [−0.76, 0.27]	0.35	NA	
Duration of Silibinin intervention (months)					
<3	1	−0.25 [−0.76, 0.27]	0.19	NA	0.26
=3	1	−2.00 [−7.89, 3.89]	0.51	NA	
>3	1	−1.00 [−3.04, 1.04]	0.34	NA	
PC-III					
Daily intake of Silibinin (g)					
<0.20	1	−0.96 [−1.43, −0.48]	<0.0001	NA	<0.0001
0.20–0.40	1	−2.05 [−2.64, −1.46]	<0.00001	NA	
>0.40	1	−2.89 [−3.63, −2.15]	<0.00001	NA	
Total intake of Silibinin (g)					
<25	1	−2.05 [−2.64, −1.46]	<0.00001	NA	<0.00001
25–50	1	−0.96 [−1.43, −0.48]	<0.0001	NA	
>50	1	−2.89 [−3.63, −2.15]	<0.00001	NA	
Duration of Silibinin intervention (months)					
<3	1	−2.89 [−3.63, −2.15]	<0.00001	NA	<0.00001
= 3	0	NA	NA	NA	
>3	2	−1.90 [−3.80, −0.01]	0.05	0	

**FIGURE 4:**
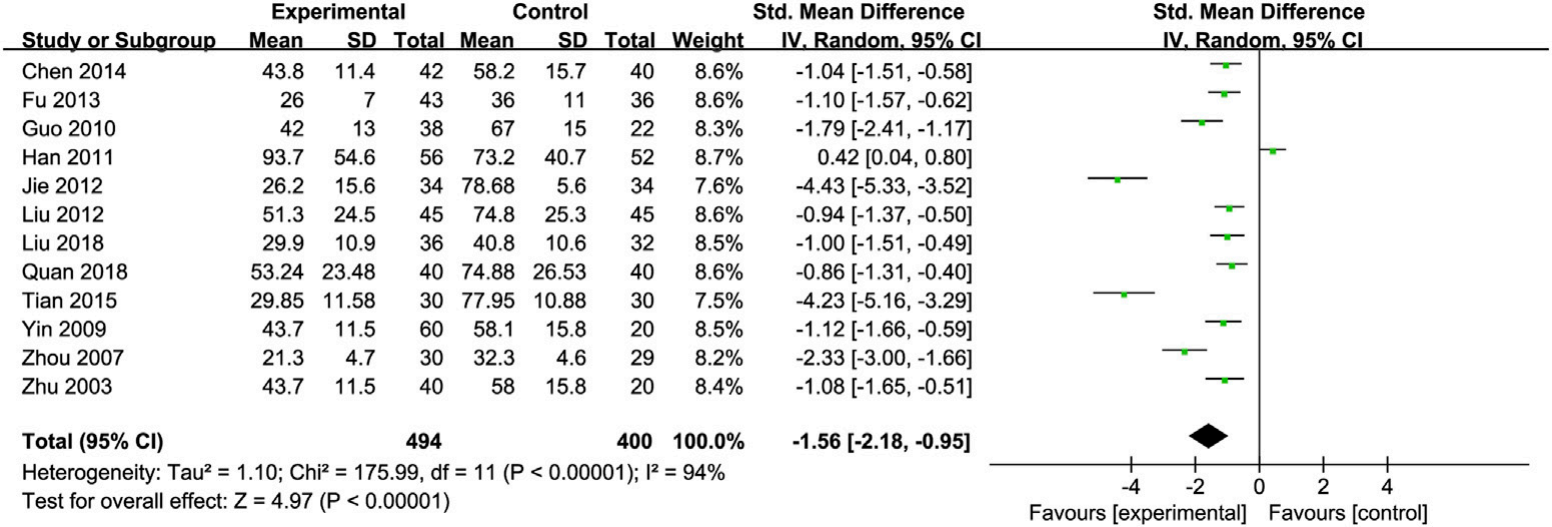
Forest plot of AST.

Aspartate Transaminase (AST): Twelve RCTs (Chen, 2014; Fu, 2013; Guo et al., 2010; Jie et al., 2012; Liu et al., 2012; Liu et al., 2018; Quan et al., 2018; Tian and Han, 2015; Yin and Yang, 2009; Zhou et al., 2008; Han et al., 2011; Zhu et al., 2003) including 494 ALD patients assessed AST levels. The reduction in AST was more pronounced in the Silibinin group compared to the control group [SMD = −1.56, 95% CI (−2.18, −0.95), P < 0.00001, I^2^ = 94%; Figure 4]. Subgroup analyses showed no significant changes in heterogeneity (Table 2).

**FIGURE 5:**
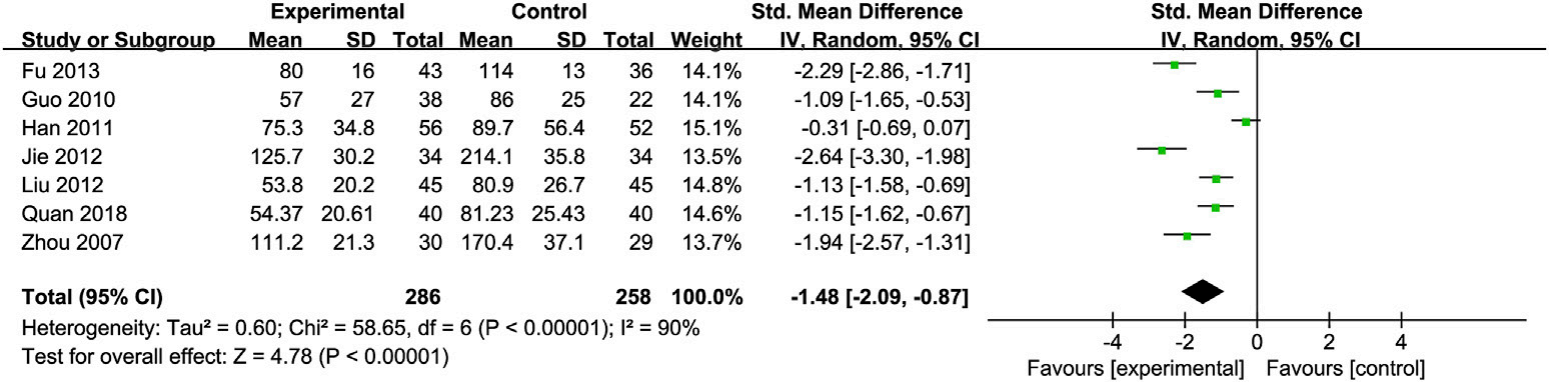
Forest plot of GGT.

Glutamyl Transpeptidase (GGT): Seven RCTs (Fu, 2013; Guo et al., 2010; Jie et al., 2012; Liu et al., 2012; Quan et al., 2018; Zhou et al., 2008; Han et al., 2011) involving 286 ALD patients evaluated GGT levels, showing a significant decrease with Silibinin intervention [SMD = −1.48, 95% CI (−2.09, −0.87), P < 0.00001, I^2^ = 90%; Figure 5]. Subgroup analysis based on intervention duration showed reduced heterogeneity (I^2^ = 25%) (Table 2).

**FIGURE 6:**
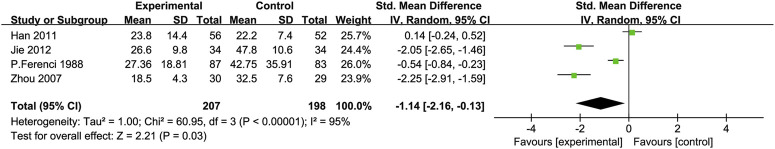
Forest plot of TBIL.

Total Bilirubin (TBIL): Four RCTs (Jie et al., 2012; Zhou et al., 2008; Han et al., 2011; Ferenci et al., 1989) with 405 participants compared TBIL levels, showing a significant difference between the experimental and control groups [SMD = −1.14, 95% CI (−2.16, −0.13), P = 0.03, I^2^ = 95%; Figure 6]. Subgroup analysis suggested reduced heterogeneity for intervention duration >3 months (I^2^ = 0%) (Table 2).

**FIGURE 7:**
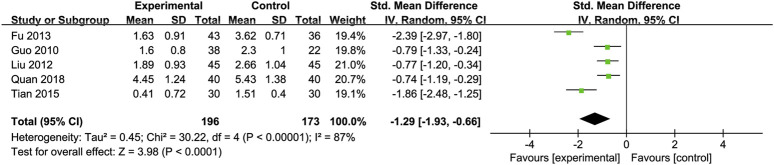
Forest plot of TG.

Triglycerides (TG): Five studies (Fu, 2013; Guo et al., 2010; Liu et al., 2012; Quan et al., 2018; Tian and Han, 2015) involving 369 participants compared TG levels, indicating Silibinin exerted an significant lipid-lowering effect [SMD = −1.29, 95% CI (−1.93, −0.66), P < 0.0001, I^2^ = 87%; Figure 7]. Subgroup analyses showed reduced heterogeneity for daily intake >0.4 g and intervention duration < 3 months (I^2^ = 0%) (Table 2).

**FIGURE 8:**
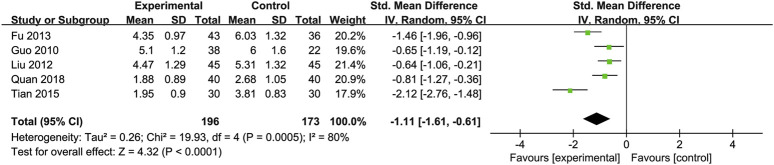
Forest plot of TC.

Total Cholesterol (TC): Five RCTs (Fu, 2013; Guo et al., 2010; Liu et al., 2012; Quan et al., 2018; Tian and Han, 2015) with 369 participants compared TC levels, showing a significant difference between the Silibinin and control groups [SMD = −1.11, 95% CI (−1.61, −0.61), P < 0.0001, I^2^ = 80%; Figure 8]. Subgroup analyses showed reduced heterogeneity for total intake 25–50 mg and intervention duration <3 months (I^2^ = 0%) (Table 2).

**FIGURE 9:**
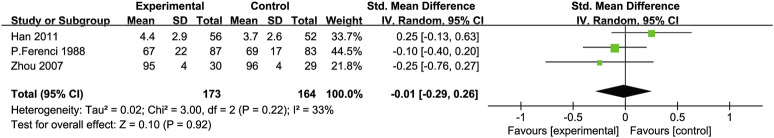
Forest plot of PT.

Prothrombin Time (PT): Three RCTs (Zhou et al., 2008; Ferenci et al., 1989; Preedy et al., 1999) with 337 participants compared PT levels, showing low heterogeneity between the experimental and control groups [SMD = −0.01, 95% CI (−0.29, 0.26), P = 0.92, I^2^ = 33%; Figure 9].

**FIGURE 10:**
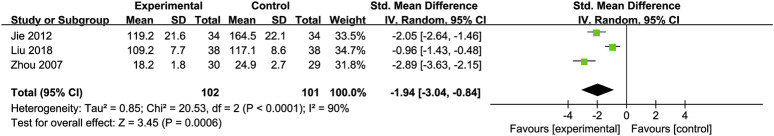
Forest plot of PC-III.

PC-III: Three RCTs (Jie et al., 2012; Liu et al., 2018; Zhou et al., 2008) with 203 participants compared PC-III levels and showed a significant reduction in PC-III levels [SMD = −1.94, 95% CI (−3.04, −0.84), P = 0.0006, I^2^ = 90%; Figure 10]. Subgroup analyses showed reduced heterogeneity (I^2^ = 0%) for intervention durations >3 months (Table 2).

**FIGURE 11:**
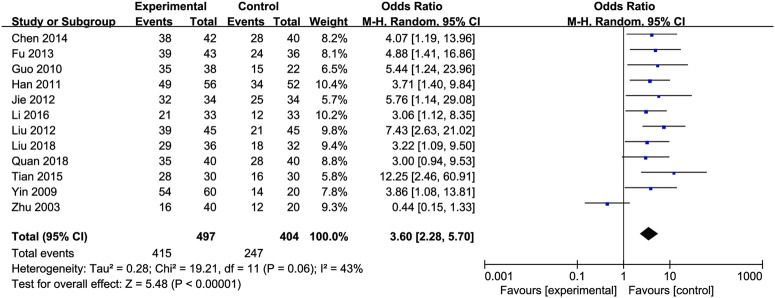
Forest plot of Effective Rate.

Effective Rate: An analysis of the effective rate was conducted on 920 participants from 12 studies (Chen, 2014; Fu, 2013; Guo et al., 2010; Jie et al., 2012; Liu et al., 2012; Li and Liu, 2016; Liu et al., 2018; Quan et al., 2018; Tian and Han, 2015; Yin and Yang, 2009; Han et al., 2011; Zhu et al., 2003). Among them, there were 502 cases in the treated group and 418 in the control group. The heterogeneity between the studies was low [OR = 3.60, 95% CI (2.28, 5.70), P < 0.00001, I^2^ = 43%; Figure 11], so we used the random-effects model.

### 3.5 Adverse events of Silibinin

Two RCTs reported adverse events, including mild alcohol withdrawal symptoms and gastrointestinal symptoms. Insomnia and nausea were the main adverse reactions. In Tian’s trial Tian and Han (2015), 2 patients in the treatment group experienced nausea, with an adverse reaction rate of 6.7%, and no adverse reactions occurred in the control group. In Liu’s study Liu et al. (2018), two patients in the experimental group experienced mild alcohol withdrawal symptoms (both insomnia), and one patient experienced mild gastrointestinal symptoms (eructation), with an adverse reaction rate of 8.3%; two patients in the control group experienced mild alcohol withdrawal symptoms (insomnia and nausea in one case each), with an adverse reaction rate of 6.3%; the difference in the incidence rates of adverse reactions between the two groups was not statistically significant. However, these symptoms were considered non-serious or harmless, and they resolved on their own after discontinuation of the medication.

### 3.6 Sensitivity analysis

**FIGURE 12:**
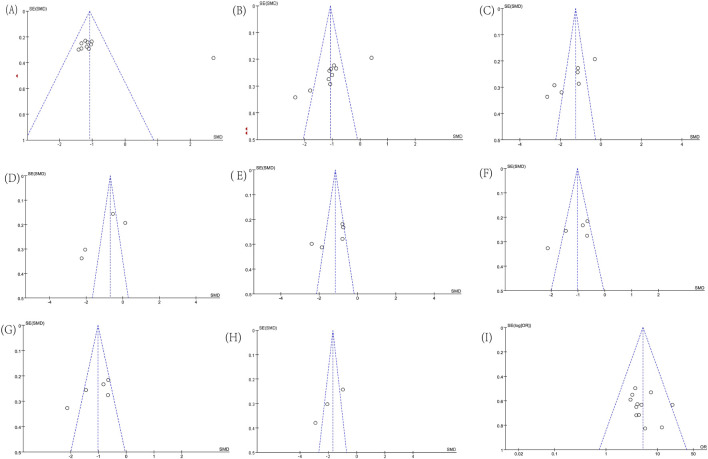
Funnel plots. **(A)** ALT; **(B)** AST; **(C)** GGT; **(D)** TBIL; **(E)** TG; **(F)** TC; **(G)** PT; **(H)** PC-III; **(I)** Effective Rate.

Funnel plots for some indicators showed asymmetry in Figures 12A–I, including ALT (Egger’s test, p = 0.051), AST (Egger’s test, p = 0.006), GGT (Egger’s test, p = 0.005), TBIL (Egger’s test, p = 0.087), TG (Egger’s test, p = 0.117), TC (Egger’s test, p = 0.038), PT ((Egger’s test, p = 0.145), PC-III (Egger’s test, p = 0.024), and Effective Rate (Egger’s test, p = 0.358). We conducted a single-factor sensitivity analysis, comparing liver function, lipids, coagulation, and liver fibrosis-related indicators, and evaluated the impact of each individual study on the heterogeneity of these indicators. The results showed that after excluding individual studies, most results did not change substantially, indicating low sensitivity and high stability of the conclusions. Specifically, after the exclusion of Tian’s study, data from 4 studies were available for the analysis of TC levels, and the results showed a significant reduction in heterogeneity (I^2^ = 58%; Supplementary Figures S1–S9).

## 4 Discussion

### 4.1 Summary of the study

In this study, we selected eight serological indicators for our analysis. Liver functions (AST, ALT, GGT, TBIL) reflected the extent of hepatocyte damage and metabolic cycle. The presence of AST/ALT >2 and elevated GGT means that the disease has progressed to the stage of alcoholic steatohepatitis (ASH). ALD is commonly characterized by intrahepatic cholestasis with predominantly elevated direct bilirubin, and a total bilirubin >85.5 μmol/L (5 mg/dL) is considered moderate alcoholic hepatitis, especially in patients with severe alcoholic hepatitis, which can be more than 20 times the upper limit of normal. These indexes can be significantly reduced after alcohol prohibition, and usually return to normal basically within 4 weeks (GGT recovery is slower), which helps the diagnosis (Guidelines for the diagnosis and treatment of alcoholic liver disease, 2010). Alcohol oxidation induces increased production of reduced coenzyme (NADH), which promotes fatty acid and triglyceride synthesis, and inhibits mitochondrial B oxidation of fatty acids, leading to elevated TC and TG, which reflects the pathological process of early alcoholic fatty liver disease (Galicia-Moreno and Gutiérrez-Reyes, 2014). PT reflects the ability of the liver to synthesise vitamin K-dependent coagulation factors. ALD patients have reduced synthesis of coagulation factors due to hepatocellular damage, leading to prolonged PT, which can be used as a sensitive indicator to monitor disease activity and prognosis. PC-III is mainly synthesised by hepatic stellate cells, and its serum level is consistent with the degree of hepatic fibrosis, which is closely related to the degree of activity of hepatic fibrosis formation. Persistent elevation of PC-III suggests that the condition of ALD may change and develop to liver fibrosis or cirrhosis, whereas the decrease of PC-III to normal may indicate remission of the condition. PC-III is not only valuable in the early diagnosis of hepatic fibrosis, but also has significance in the prognosis of chronic liver disease (Torkadi et al., 2014).

The systematic review of 15 RCTs involving 1,221 patients showed that Silibinin, as an adjuvant therapy, can improve liver function, lipid abnormalities, liver fibrosis indicators, and coagulation function in ALD patients, with minimal adverse reactions. There was a high degree of heterogeneity in multiple results, such as ALT, AST, GGT, which reduced the reliability of the pooled estimates. Therefore, we made the following analyses of the sources of heterogeneity: (1) Differences in study designs: most of the included studies were observational, making it difficult to completely exclude potential confounders. Although some of the studies controlled for some confounding variables, not all relevant factors were considered, and the diagnostic criteria and methods of measuring ALD-related indicators varied across included studies, which may have reduced the reliability and comparability of results. Eight RCTs (Fu, 2013; Guo et al., 2010; Liu et al., 2012; Li and Liu, 2016; Liu et al., 2018; Quan et al., 2018; Tian and Han, 2015; Han et al., 2011) used uniform diagnostic criteria (Zeng et al., 2010): ① Alcohol consumption history: A history of chronic alcohol consumption, usually over 5 years, with an equivalent amount of ethanol >240 g/d for men and >20 g/d for women, or a history of heavy alcohol consumption within 2 weeks, with an equivalent amount of ethanol >80 g/d. ② Symptoms: Clinical symptoms are non-specific, may be asymptomatic, or right upper abdominal distension and pain, loss of appetite, fatigue, weight loss, jaundice. With the aggravation of the disease, there may be neuropsychiatric symptoms, spider nevus, liver palms and so on. ③ Laboratory tests: AST, ALT, GGT, TBiL, PT, carbohydrate-deficient transferrin (CDT), mean plasma volume (MCV) increased. These indicators may decrease significantly after abstinence from alcohol and usually return to normal within 4 weeks, and AST/ALT >2. ④ Imaging examinations: Liver B-ultrasound or CT with typical signs, for example, diffuse hepatic hypodensity, where the ratio of the CT values of the liver to the spleen is less than or equal to one. ⑤ Exclusion of current hepatophilic virus infection as well as drugs, toxic liver injury and autoimmune liver disease. Two RCTs (Yin and Yang, 2009; Zhou et al., 2008; Han et al., 2011; Zhu et al., 2003) used different diagnostic criteria. In the history of alcohol consumption, it was stated that the daily alcohol consumption was more than 40 g, but the daily alcohol consumption criteria for different genders were not specified, and the laboratory indexes only mentioned abnormal liver functions (AST, ALT) and dyslipidemia (TG, TC), CDT, MCV not involved. The other two trials (Jie et al., 2012; Liu et al., 2012; Li and Liu, 2016; Liu et al., 2018; Quan et al., 2018; Tian and Han, 2015; Yin and Yang, 2009; Zhou et al., 2008) also had the same problem of a single laboratory test, with only ALT, AST and GGT increasing significantly in the serologic indexes. Yet 3 others (Chen, 2014; Fehér et al., 1998; Ferenci et al., 1989) did not specify specific diagnostic criteria. This meta-analysis included studies from different years, so the diagnostic criteria referenced in the disease guidelines were different, and these minor changes led to heterogeneity in the results. (2) Dosage variations: the lack of systematic assessment of the severity of ALD prevented exploration of the relationship between different doses and its efficacy. (3) Baseline patient characteristics and demographic factors: different races, ages, and genders respond differently to medications, which has an impact on study results. Based on this, subgroup analyses and sensitivity analyses were performed to check the reliability of the results and to improve the precision.

In this subgroup analysis, increasing the intake of Silibinin did not have a significant effect on improving liver function. However, prolonged dosing, especially for more than 3 months, may be more conducive to lowering aminotransferase levels. Conversely, we found that increasing the intake of Silibinin can enhance its effect on lipids. Therefore, based on these results, long-term intake of lower doses of Silibinin is recommended to bring greater benefits to ALD patients in a clinical setting.

### 4.2 The possible therapeutic mechanisms of ALD

ALD is defined as liver damage caused by chronic excessive alcohol consumption, whose complex mechanisms include: (1) Toxic effects of alcohol in the body: Ethanol is converted into acetaldehyde in the liver after metabolism, damaging the microtubule system in liver cells, leading to hepatocellular steatosis or even apoptosis, and damaging mitochondrial fatty acid beta-oxidation, among other toxic effects (Lim et al., 2003; Preedy et al., 1999). (2) Generation of free radicals and lipid peroxidation: Ethanol metabolism in the liver generates a large number of peroxyl radicals, hydroxyl radicals, and hydroxyethyl radicals, leading to enhanced membrane lipid peroxidation, formation of lipid radicals and peroxides, disrupting normal membrane structure, and directly or indirectly acting as oxidants, causing damage to membrane components, abnormal mitochondrial function, and lipid deposition in the liver (Jeon and Carr, 2020). (3) Immune response and cytokines: The liver’s immune system is mainly composed of the innate immune system. Studies have reported that the occurrence of ALD involves multiple cytokines such as TNF-α, IL-1, IL-6, IL-8, TGF-β, etc., with significantly elevated expression levels, indicating a severe inflammatory response in the liver, stimulating stellate cells to transform into fibroblasts, leading to liver fibrosis (Xiang et al., 2020; Arab et al., 2020). (4) Endotoxin action: Excessive ethanol induces increased intestinal permeability to endotoxins, mediated by PLA2-mediated membrane phospholipid degradation and induction of free radicals, activating Kupffer cells to release neurotransmitters, leading to liver cell damage (Han et al., 2015). (5) Ferroptosis: Excessive drinking promotes intestinal epithelial cell iron absorption and liver cell iron uptake, exacerbating ALD liver damage (Preedy et al., 1999). (6) Long-term excessive drinking damages the intestinal mucosal barrier, leading to excessive translocation of bacteria, bacterial components, and endotoxins to the liver, causing liver inflammation and fibrosis (Liu et al., 2020; Avila et al., 2020).

ALD is caused by a single pathogenic factor but has complex mechanisms involving fatty degeneration, inflammation, fibrosis, and carcinogenesis, resulting from the combined effects of susceptibility genes, gut microbiota, oxidative stress damage, immune damage, and programmed cell death, among other factors (Louvet and Mathurin, 2015).

Among various bioactive ingredients, Silymarin is an active agent. It is a standard mixture of flavonoid compounds (70%–80%) with Silybin, Silibinin, and Silibinin diaminocyclohexane, with Silibinin being the main active chemical component (Loguercio and Festi, 2011). Silymarin is used to treat liver diseases, including acute and chronic hepatitis, hepatitis/drug-induced hepatitis, cirrhosis, and toxic liver disease. Alcoholic liver damage is associated with changes in the oxidative capacity of cells caused by ethanol metabolism. Activation of secondary ethanol metabolic pathways (such as the microsomal ethanol oxidation system (MEOS), high levels of lipids, and increased intracellular oxidative stress cause lipid peroxidation, leading to the disruption of cellular and mitochondrial membranes and cell death (Galicia-Moreno and Gutiérrez-Reyes, 2014).

Song et al. showed that Silymarin (200 mg/kg body weight) in mice reduced oxidative stress as evidenced by oral ethanol at 5 g/kg body weight every 12 h, three times the total amount, and prevented the increase in ALT, depletion of glutathione (GSH), lipid peroxidation, and increased TNF-α (Song et al., 2006). Silybinin and SilPho optimize mitochondrial metabolism and the electron transport chain, increase intracellular superoxide dismutase SOD activity, and reduce monoamine oxidase (MAO) activity, ultimately reducing intracellular ROS levels, thereby improving mitochondrial function, reduceing transaminase levels, such as ALT, AST, GGT (Galicia-Moreno and Gutiérrez-Reyes, 2014; Das and Mukherjee, 2012). Some studies have found the antioxidant effects of silymarin to be effective in treating neurological disorders as well. It is demonstrated that silymarin and silybin improve neurogenesis in the prefrontal cortex and hippocampus by modulating SOD and catalase (CAT) activities, alleviating symptoms of depression and anxiety (Rostamian et al., 2023).

Extensive researches have shown that silymarin suppresses the expression of pro-inflammatory compounds (Wadhwa et al., 2022). Given that silymarin is rich in silybin, it plays a role in lowering serum inflammatory cytokine levels. Furthermore, silymarin also prevents leukocyte migration and the production of neutrophils at inflammation sites (Khazaei et al., 2022). This effect is linked to its ability to stabilize cell membrane integrity, decrease the release of inflammatory mediators, and inhibit the activities of cyclooxygenase and lipoxygenase. Silibinin has been found to reduce lipid inflammatory responses by regulating inflammatory mediators such as TGF-β and IL-4/IL-10, ameliorating lipid metabolism-related diseases (Surai et al., 2024). Additionally, studies have found that Silibinin also has anti-inflammatory effects on NAFLD. For example, Zhang et al. (2018) observed that Silymarin significantly inhibited the activation of NLRP3 inflammasomes in NAFLD by increasing NAD^+^ levels, preserving the NAD^+^-dependent α-tubulin deacetylase sirtuin (SIRT) 2 activity, and inhibiting acetylated α-tubulin-induced NLRP3 inflammasome activation, demonstrating the anti-inflammatory and liver-protective effects of Silibinin mainly focused on cytokine release (Zhang et al., 2018).

Excessive alcohol consumption leads to decreased insulin sensitivity, inhibits the hepatic insulin signaling pathway, and disrupts glucose and lipid metabolism, which are important mechanisms in the pathogenesis of ALD (Cheng et al., 2019). Alcohol affects many aspects of hepatic lipid metabolism. Pathways leading to hepatic cell lipid droplet accumulation include alcohol-induced hepatic fatty acid uptake, oxidative damage to fatty acids, promotion of lipid neosynthesis and neutral lipid storage, and inhibition of lipid production and decomposition metabolism (Jeon and Carr, 2020). Studies have shown that alcohol-induced hepatic steatosis is accompanied by upregulation of lipid droplet-stabilizing proteins, adipose differentiation-related proteins (ADRP), as well as genes related to fatty acid synthesis, fatty acid synthase (FASN), and acetyl-CoA carboxylase (ACACA), exacerbating the condition (Levin et al., 2012).

The increase in hepatic stellate cells (HSC) and Kupffer cells is considered a decisive factor in liver fibrosis (Surai et al., 2024). Experimental studies have shown that Silymarin impairs HSC proliferation, prevents their transformation into myofibroblasts, and downregulates gene expression of extracellular matrix components required during fibrosis (Clichici et al., 2015; Fuchs et al., 1997). In a research Li et al. (2018), discovered that both silymarin and silybin play a significant role in stimulating nerve growth. Their findings indicated that these compounds elevate the levels of Brain-derived neurotrophic factor (BDNF), a crucial protein that supports the survival and proliferation of neurons. Through this mechanism, silymarin and silybin promote the growth of neural stem cells by activating the BDNF/TrkB signaling pathway. Notably, this pathway shares certain characteristics with the regenerative processes seen in liver cells, or hepatocytes, suggesting a broader applicability of these findings in understanding neural regeneration and repair.

**FIGURE 13:**
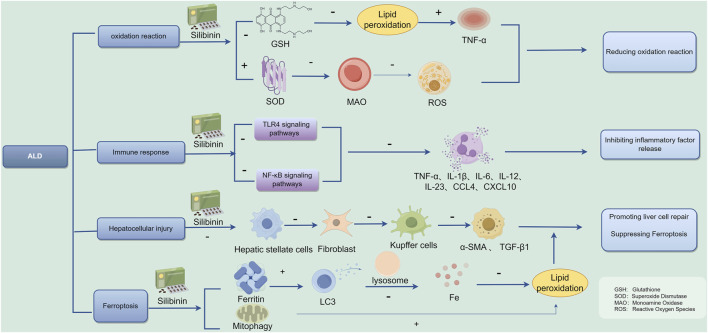
Possible therapeutic mechanisms of ALD. “+” = promote, “−” = inhibit.

Numerous preclinical *in vitro* and *in vivo* studies have effectively demonstrated potential molecular targets of Silibinin for anticancer effects. Indeed, Silibinin can interfere with tumor induction processes by modulating inflammation cascades and reducing the toxic potential of ROS genes (Brandon-Warner et al., 2012). The current study shows that Silymarin is effective in reducing transaminases during viral hepatitis. However, with regard to histological or serum viral loads, its use had no direct effect (Mayer et al., 2005). Lah et al. (2007) demonstrated that Silibinin effectively inhibits proliferation and promotes apoptosis in human hepatocellular carcinoma cells by targeting specific cell cycle protein kinases and activating genes responsible for encoding Caspase 3–9. Ferroptosis is involved in the pathogenic process of ALD, and inhibition of ferroptosis: is a potential therapeutic target for ALD treatment. In a study conducted by Song et al. (2022), it was discovered that Silibinin has the ability to prevent ferroptosis induced by acetaldehyde in liver cells such as HepG2 and HL7702. This finding suggests that Silibinin could be a promising treatment option for individuals suffering from ALD, and the possible mechanistic map of Silibinin for the treatment of ALD is summarized in Figure 13. ALD is a serious health condition that can lead to various complications if left untreated. With the potential of Silibinin to inhibit ferroptosis, a process known to contribute to liver damage, there is hope for a new therapeutic approach to managing this disease. Further research and clinical trials may be needed to validate the effectiveness of Silibinin as a treatment for ALD, but the initial results are promising and offer a glimmer of hope to individuals battling this condition.

Silybin has antioxidant, anti-inflammatory, antifibrotic, and protective effects on liver cell membranes and organelles, and its mechanism of action is distinct for different degrees of alcoholic liver disease. For mild ALD and alcoholic fatty liver disease, silybin can play the role of antioxidant and anti-lipid peroxidation, prevent or reduce the steatosis of hepatocytes and improve liver function (Medina and Moreno-Otero, 2005). For moderate alcoholic liver disease, in addition to antioxidant, silybin can regulate the metabolism of hepatocytes, promote the repair of damaged hepatocytes, and at the same time, it can inhibit inflammatory reactions, reduce the infiltration of inflammatory cells in liver tissue, and prevent further development of inflammation leading to liver fibrosis (Zhang et al., 2013). In the severe stage of alcoholic liver disease, silymarin can also play a somewhat positive role. Although it cannot completely reverse serious lesions such as cirrhosis that have already developed, it can slow down the process of liver fibrosis. It can be used in combination with other drugs, such as glucocorticoids, metadoxine, and adenosylmethionine, to improve the overall function of the liver, improve the quality of life of patients, and prolong survival. Five published clinical studies on the application of silibinin in the treatment of ALD have enrolled more than 600 patients with alcoholic cirrhosis (Aghazadeh et al., 2011; Trinchet et al., 1989; Bunout et al., 1992). The results showed that after a mean of 41 months of treatment, the 4-year cumulative survival rate of patients in the silymarin-treated group was significantly higher than that of the placebo group, and subgroup analyses suggested that the efficacy of silymarin in alcoholic cirrhosis was independent of the severity of liver disease (Parés et al., 1998).

### 4.3 Safety

Pharmacological and clinical experiments have demonstrated the good safety profile of Silibinin capsules. General safety indices, such as blood pressure, pulse rate, blood biochemical indices and electrocardiogram, showed no abnormality. The drug approved mainly for the recovery of abnormal liver function in acute and chronic hepatitis and fatty liver, and have been recommended for use in several therapeutic guidelines. Through the study of different liver drug enzymes CYP, it is also proved that silymarin will not interact with other drugs, and can be combined with antiviral drugs, liver-protecting drugs and lipid-lowering drugs in the clinic, which can enhance the efficacy of liver-protecting and enzyme-lowering. It should be noted that the current trials do not include all groups of people, the safety of some special groups has not yet been determined, so pregnant females, lactating women and persons allergic to this drug should be used with caution (Guo et al., 2014).

Silibinin, an important active component of the flavonolignan complex Silymarin, has been shown to protect liver cells through various pharmacological pathways such as enhancing hepatocyte membrane stability, antioxidation, antifibrosis, and anti-inflammation (Kazazis et al., 2014). It has a well-established pharmacological mechanism and wide clinical application as a hepatoprotective agent. In clinical trials, it has a broad range of indications, with few serious adverse reactions, occasional reports of insomnia, According to a Meta-analysis by Zheng et al. (2022), the incidence of adverse reactions to silymarin capsules was low, about 7% overall, and not significantly different from the control group. Some clinical studies have shown that individuals may experience nausea, dyspepsia, or mild diarrhea when taking more than 1,500 mg per day, though all are typical gastrointestinal reaction and symptoms disappear after discontinuing the dose (Leng-Peschlow, 1996). Data from a 2017 clinical trial showed that patients were using silymarin 2,100 mg/day for 48 weeks and results showed that silymarin and silymarin were well-tolerated (Wah Kheong et al., 2017). Special attention should be paid to the fact that people who are allergic to the Asteraceae plant need to be cautious, and the possibility of adverse reactions such as anaphylaxis cannot be ruled out (Tamayo and Diamond, 2007). In our meta-analysis, the daily intake of Silibinin capsules ranged from 0.14 g to 0.42 g, with minimal adverse reactions, all of which were mild. Silibinin is rapidly absorbed from the stomach and distributed throughout the body, primarily metabolized and eliminated through glucuronidation, with distribution and elimination half-lives of 0.7 ± 0.4 and 2.4 ± 1.1 h, respectively (Duan et al., 2011). It has hepatotropic properties and is widely used to treat viral hepatitis, ALD, metabolic liver disease, liver cancer, and common liver diseases, including cirrhosis and HCC, indicating its significant biological role (Hackett et al., 2013).

### 4.4 Quality of evidence

We evaluated the results of 15 RCTs covering a wide patient population for each outcome. Our study showed consistent benefits of Silibinin on several key endpoints, including liver functions (AST, ALT, GGT, TBIL), lipid levels (TG, TC), coagulation indicators (PT), liver fibrosis indicators (PC-III), and improvement in effectiveness, with SMD ranging from moderate to significant (0.01–1.69). This study utilized Cochrane systems, Review Manager 5.4.1, and STATA 14.0 for analysis, comprehensively and objectively evaluating its clinical efficacy and safety, all of which showed strong confidence levels, reinforcing the potential of Silibinin capsules as an important therapeutic intervention for ALD.

### 4.5 Limitations of this study

However, the potential of Silibinin capsules for ALD needs to be approached with caution because of some significant limitations inherent in this systematic review. Firstly, this study involved 968 participants, mainly from China, so the general applicability of the drug is limited. The efficacy of silibinin related to the genetics, environment and lifestyle of different human races, as well as differences in medical systems and treatment protocols across countries, which limit the applicability of the study results to different populations and settings. Secondly, some included studies did not describe blinding and allocation concealment, and several trials showed unclear or high-risk biases, which may have caused selection bias and measurement bias, reducing the reliability of the results. Finally, the inconsistency in diagnostic criteria, duration of treatment and dosage of the included studies, lacking long-term follow-up data to assess sustained efficacy and safety, and most were small-sample studies, limiting the ability to draw correct conclusions regarding drug efficacy and safety. It is necessary to emphasize the need for future RCTs to validate the findings in broader, more diverse populations.

## 5 Conclusion

This systematic review offered primary evidence that Silibinin capsules may be an effective adjunctive therapy to improve liver function, lipid levels, coagulation time, fibrosis degree, and clinical effectiveness in patients with ALD, offering an effective option for clinical ALD treatment strategies. Currently, the mechanism of action of this drug in treating ALD is not clear, and we look forward to more experimental studies to demonstrate the clinical value of Silibinin capsules.

## Data Availability

The original contributions presented in the study are included in the article/Supplementary Material, further inquiries can be directed to the corresponding author.
