# Supplementary figures and images for: Evidence construction of Silibinin capsules against alcoholic liver disease based on a meta-analysis and systematic review
Source: Front Pharmacol. 2025 Feb 4;16:1516204. doi: 10.3389/fphar.2025.1516204 (PMC11832396; doi:10.3389/fphar.2025.1516204)

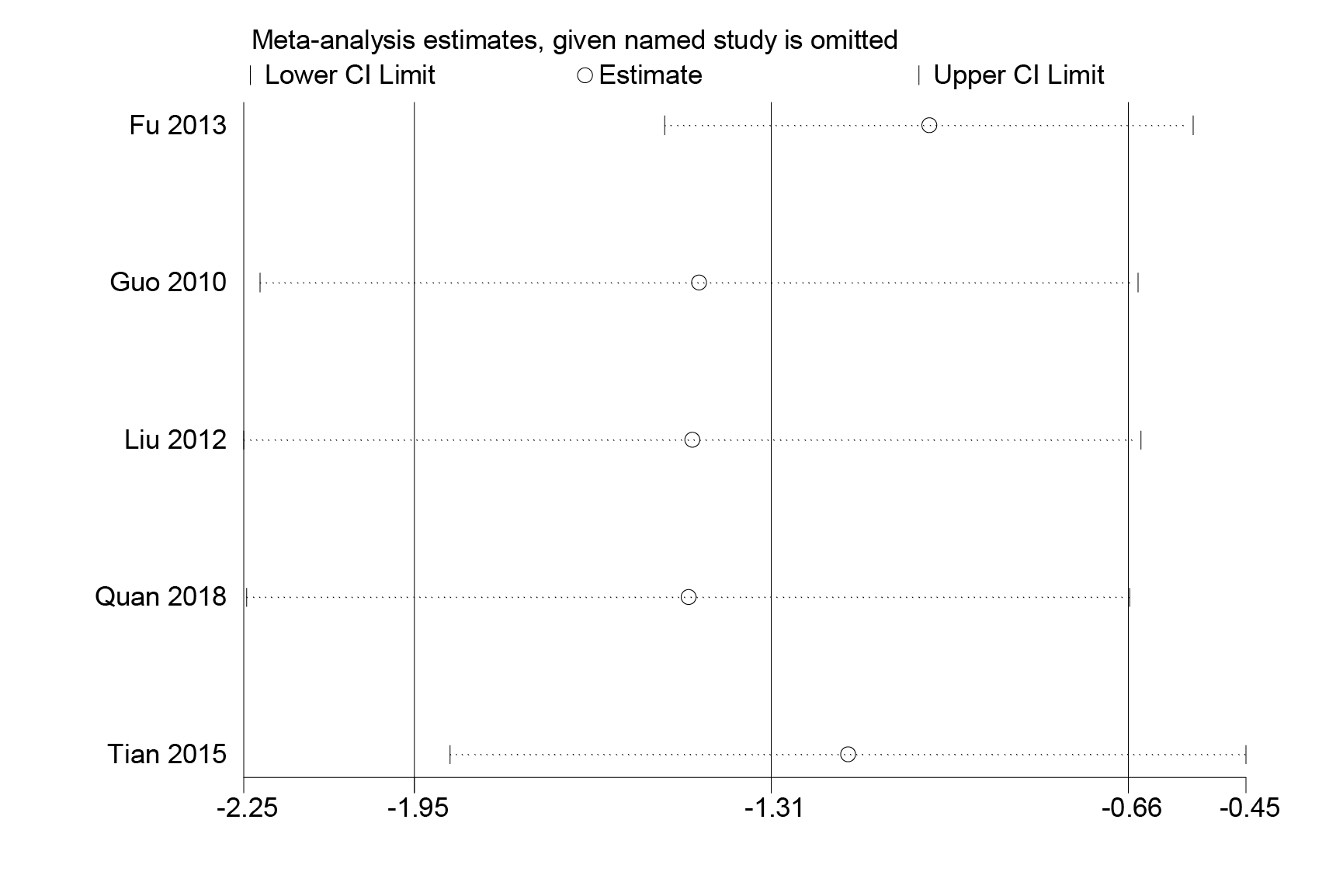

Supplement: Supplementary file 1 [file Image5.PNG]

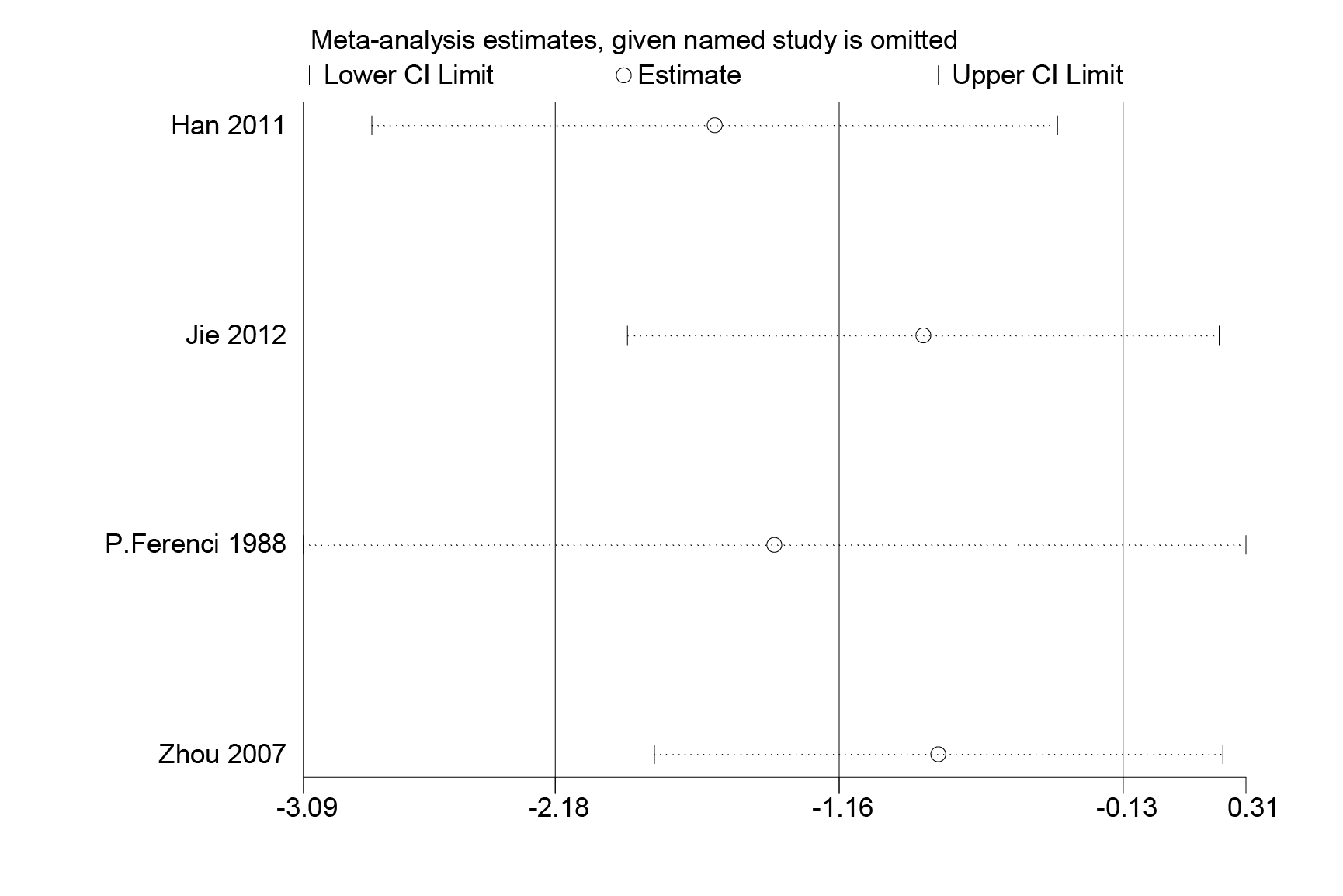

Supplement: Supplementary file 2 [file Image4.PNG]

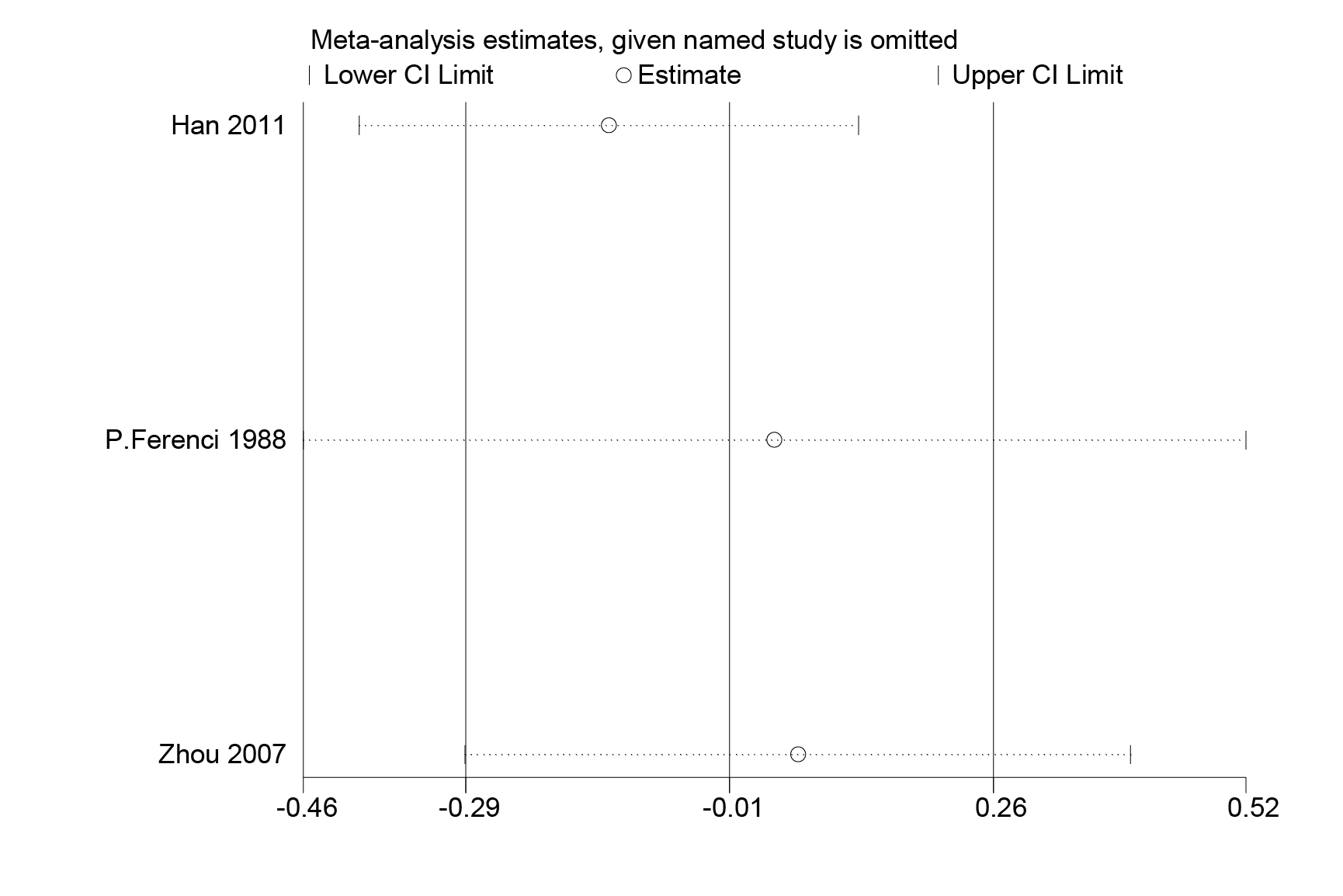

Supplement: Supplementary file 3 [file Image7.PNG]

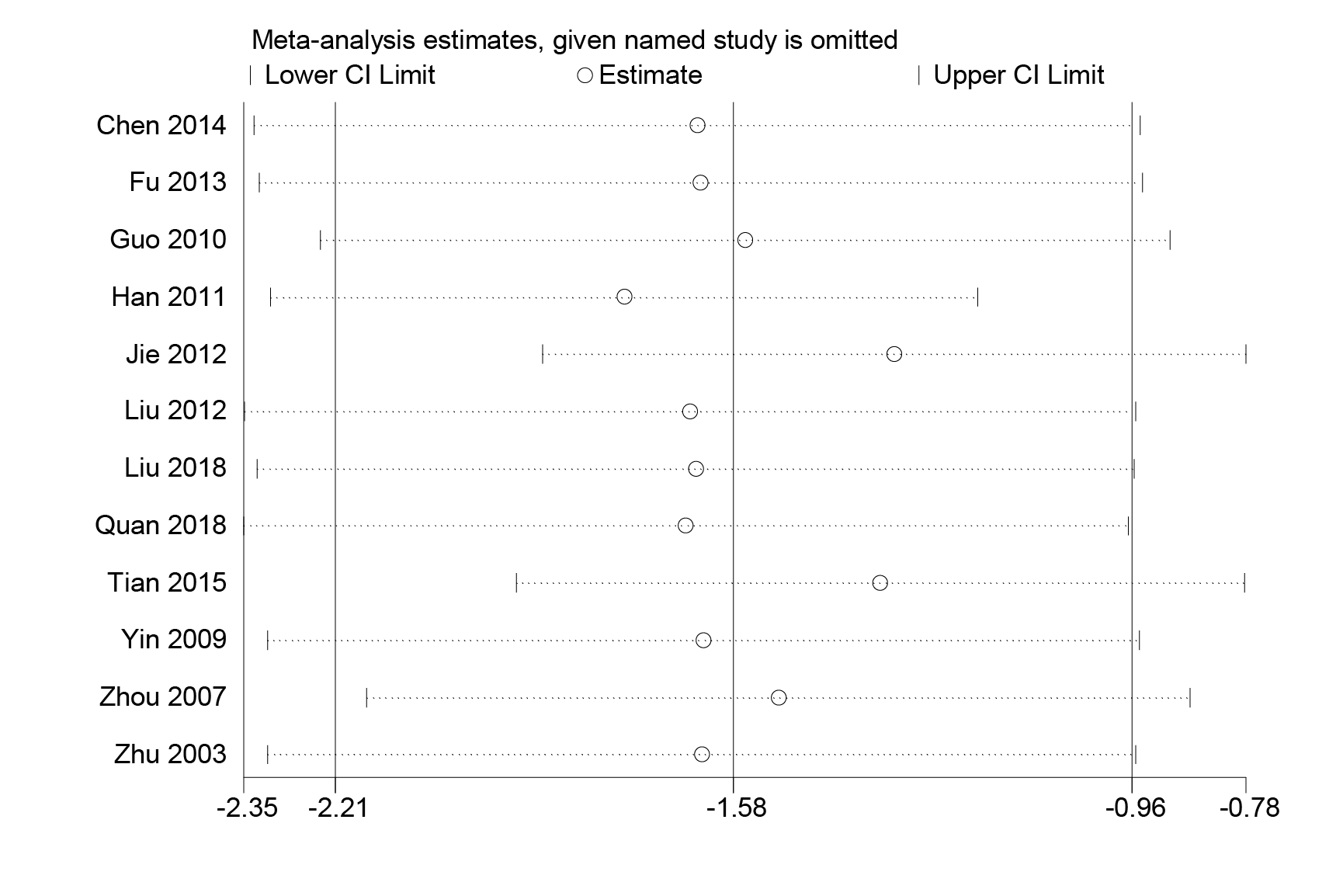

Supplement: Supplementary file 4 [file Image2.PNG]

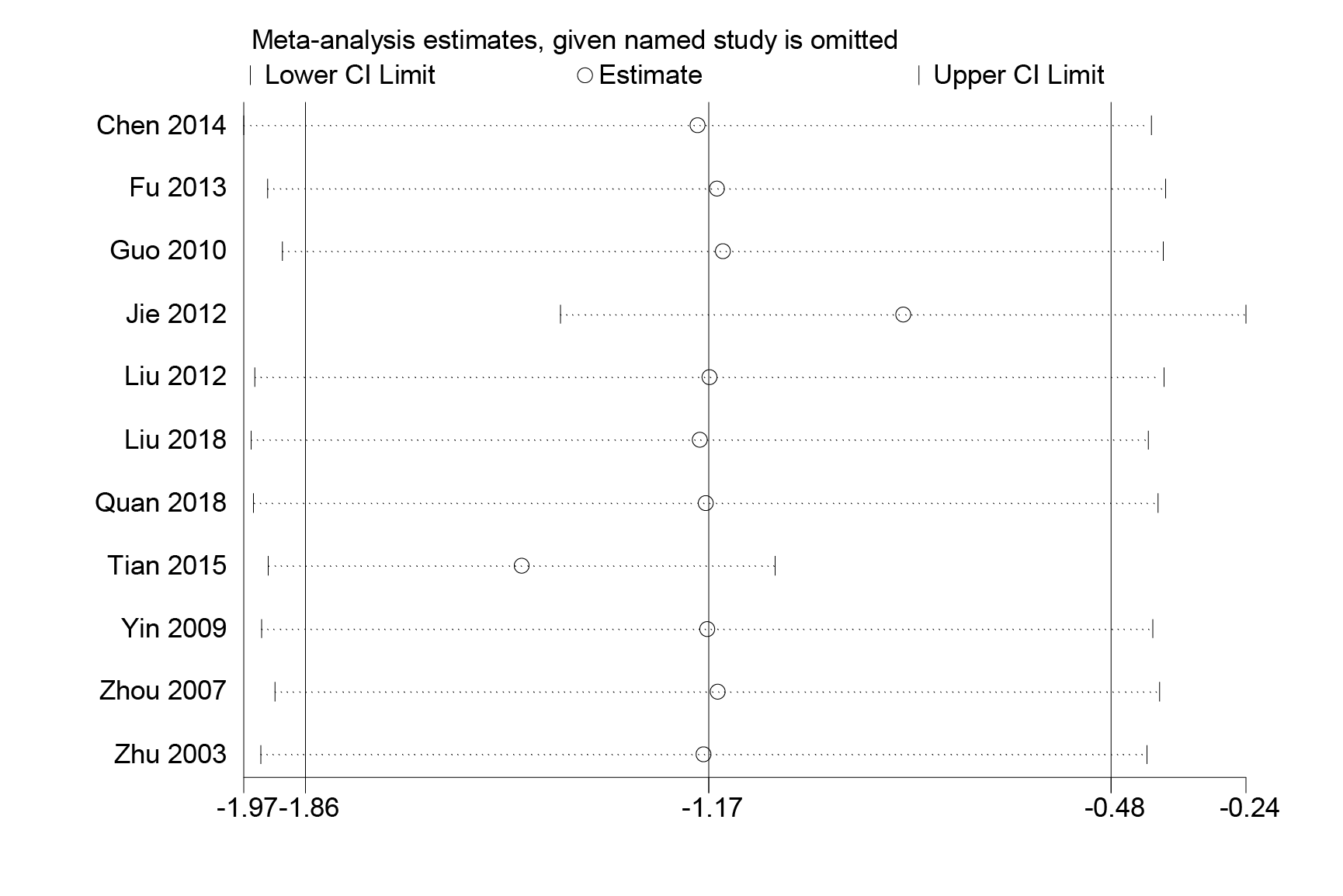

Supplement: Supplementary file 5 [file Image1.PNG]

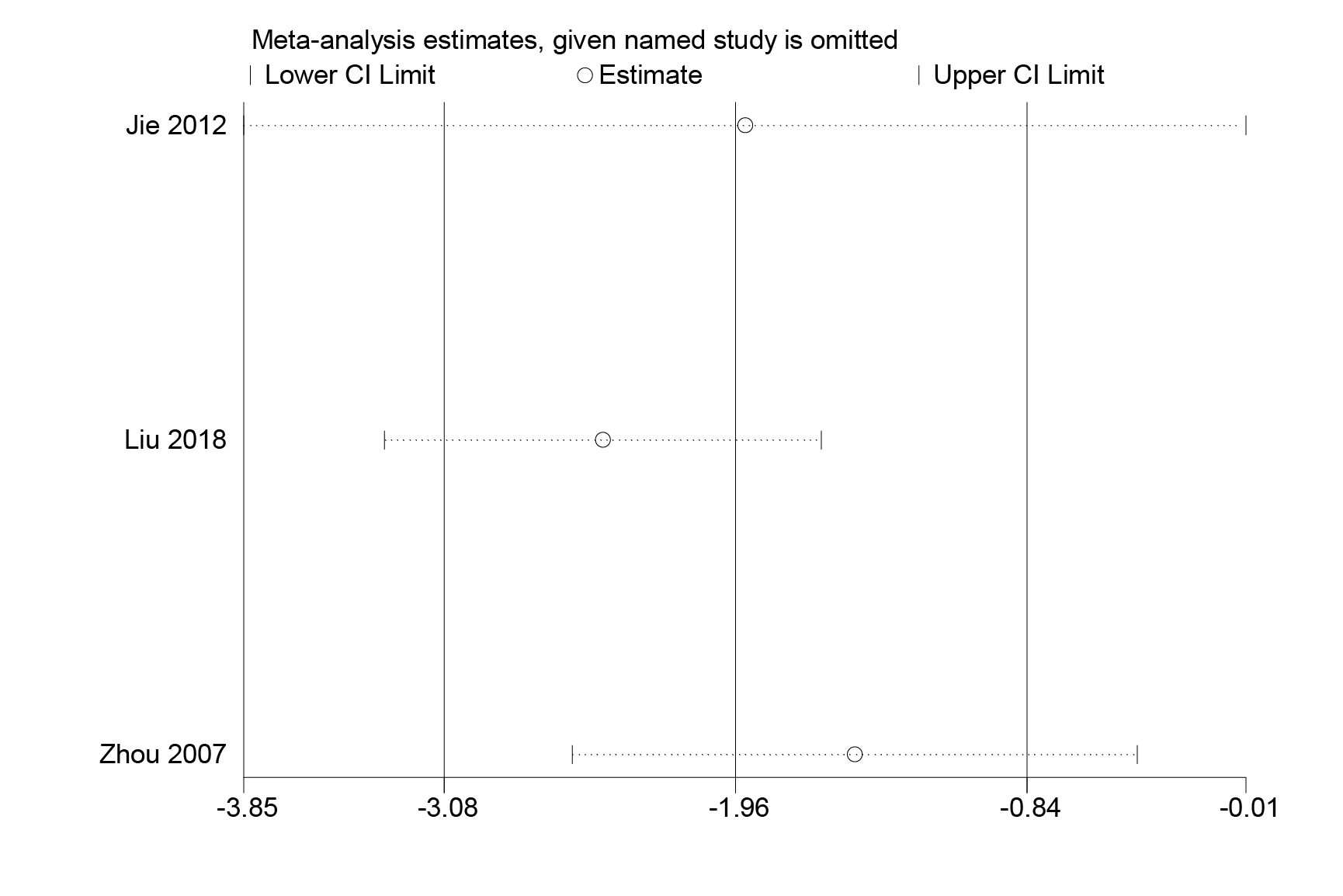

Supplement: Supplementary file 6 [file Image8.PNG]

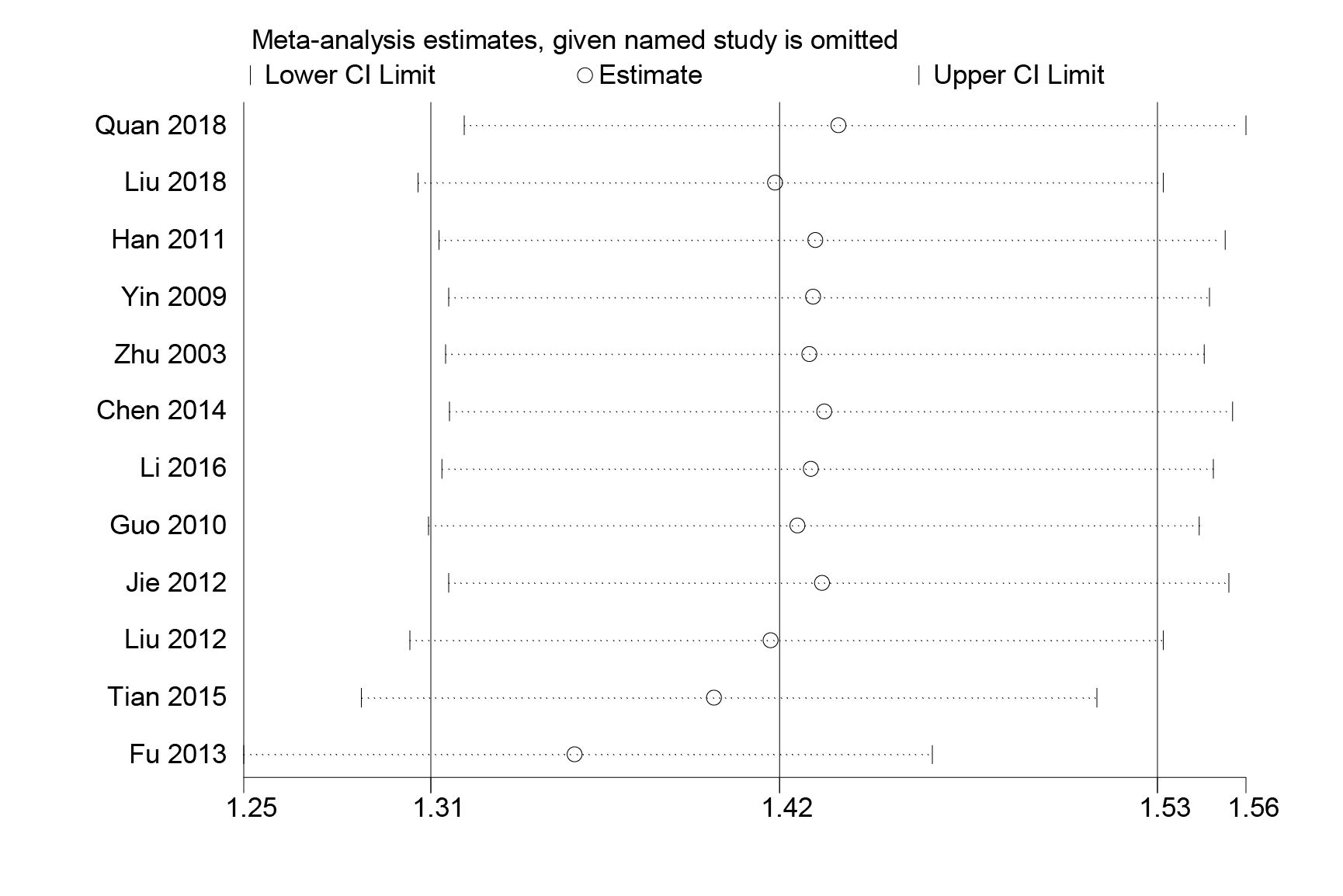

Supplement: Supplementary file 7 [file Image9.PNG]

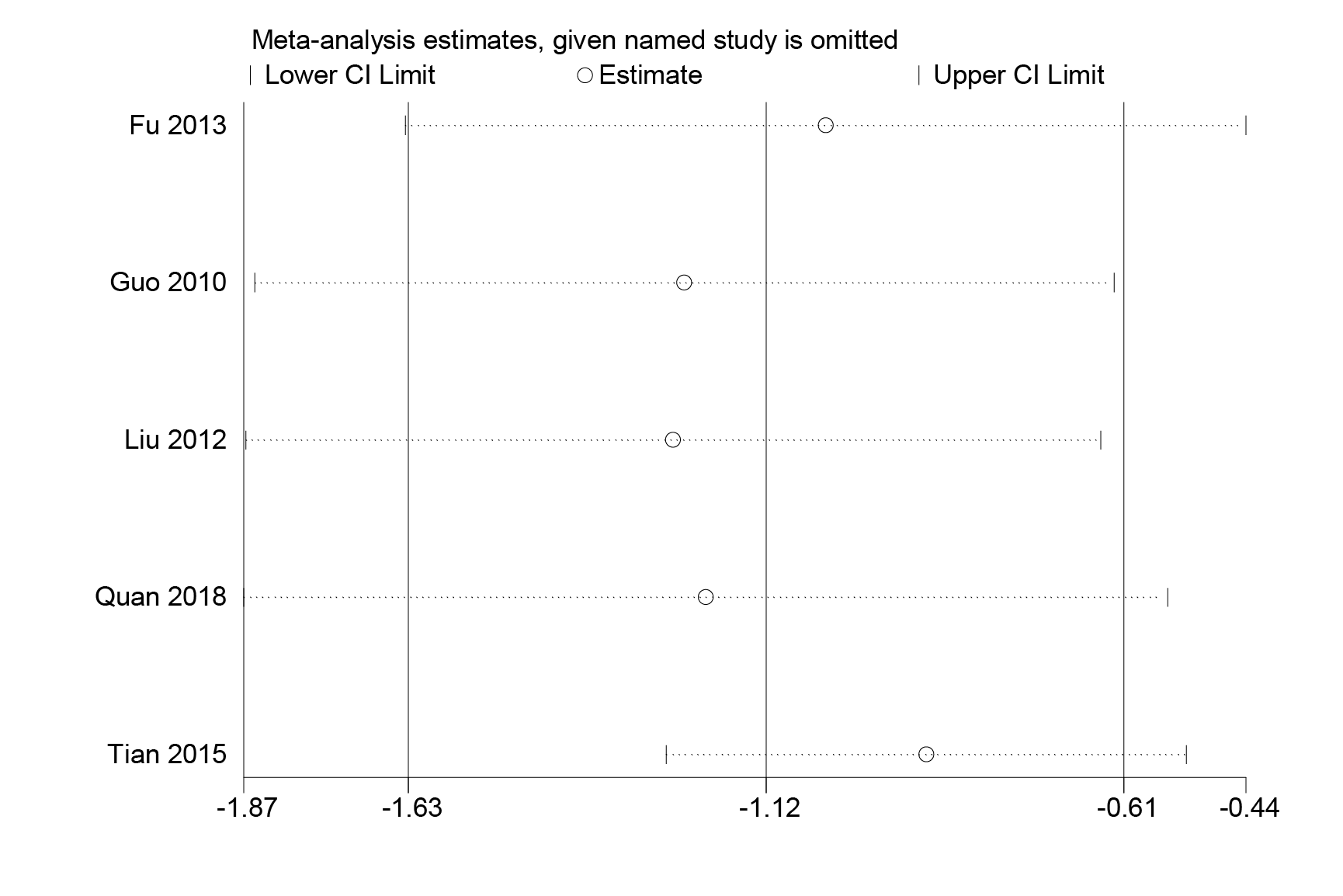

Supplement: Supplementary file 8 [file Image6.PNG]

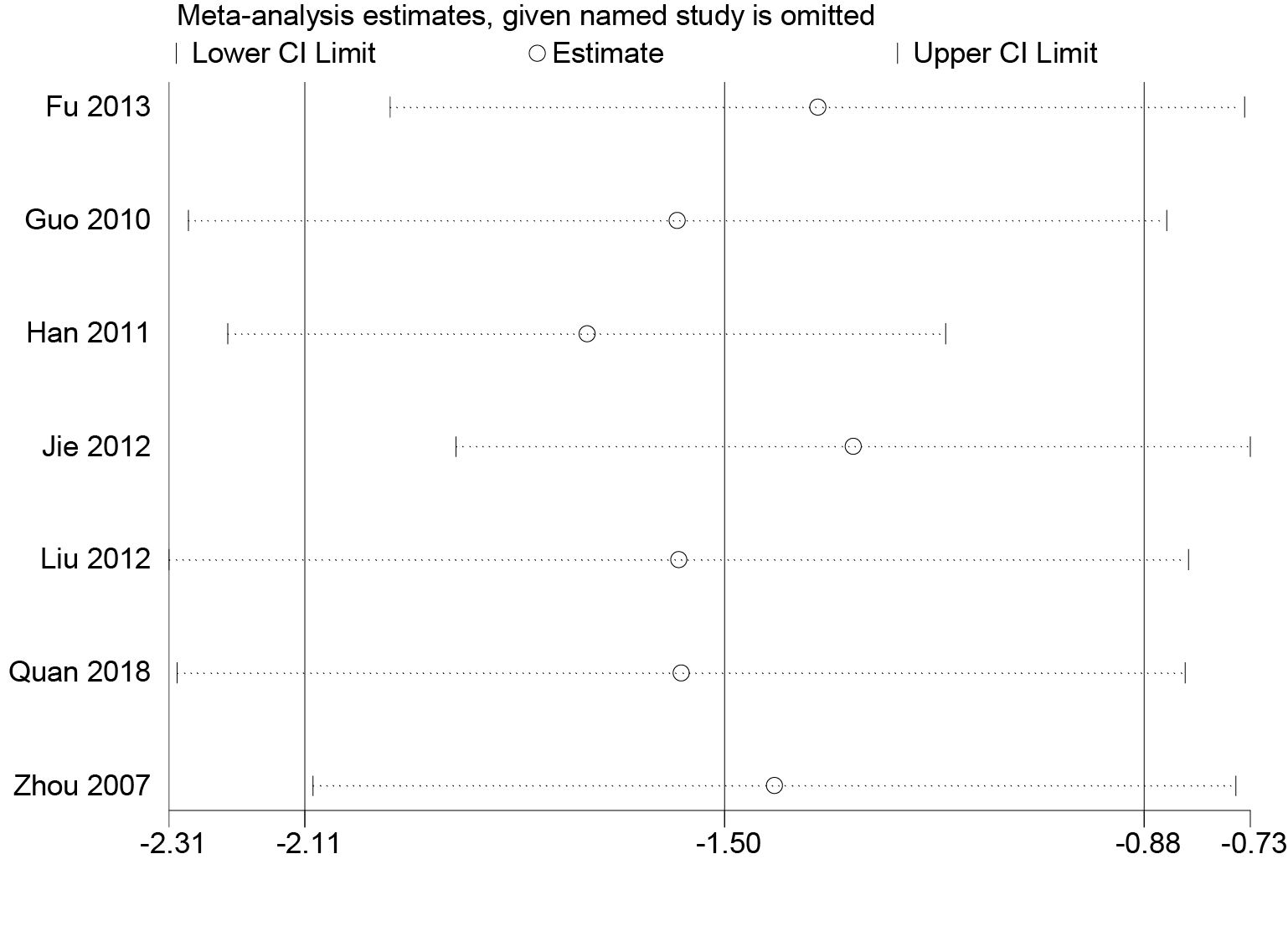

Supplement: Supplementary file 9 [file Image3.PNG]
